# Development of Enriched Core Competencies for Health Services and Policy Research

**DOI:** 10.1111/1475-6773.12847

**Published:** 2018-03-11

**Authors:** Stephen Bornstein, Melissa Heritage, Amanda Chudak, Robyn Tamblyn, Meghan McMahon, Adalsteinn D. Brown

**Affiliations:** ^1^ Institute of Health Policy, Management and Evaluation Dalla Lana School of Public Health University of Toronto Toronto ON Canada; ^2^ Li Ka Shing Knowledge Institute St. Michael's Hospital Toronto ON Canada; ^3^ Department of Obstetrics and Gynecology University of Toronto Toronto ON Canada; ^4^ Dalla Lana School of Public Health University of Toronto Toronto ON Canada; ^5^ Department of Political Science and Division of Community Health and Humanities Memorial University St. John's NL Canada; ^6^ Newfoundland and Labrador Centre for Applied Health Research St. John's NL Canada; ^7^ Independent Management Consultant Toronto ON Canada; ^8^ Institute of Health Services and Policy Research Canadian Institutes of Health Research Montreal QC Canada; ^9^ Department of Epidemiology, Biostatistics, and Occupational Health McGill University Montréal QC Canada; ^10^ Institute of Health Policy, Management and Evaluation University of Toronto Toronto ON Canada

**Keywords:** Health services research, graduate training modernization, competency‐based education, research personnel, health services research impact

## Abstract

**Objective:**

To develop an enriched set of core competencies for health services and policy research (HSPR) doctoral training that will help graduates maximize their impact across a range of academic and nonacademic work environments and roles.

**Data Sources/Study Setting:**

Data were obtained from multiple sources, including literature reviews, key informant interviews, stakeholder consultations, and Expert Working Group (EWG) meetings between January 2015 and March 2016. The study setting is Canada.

**Study Design:**

The study used qualitative methods and an iterative development process with significant stakeholder engagement throughout.

**Data Collection/Extraction Methods:**

The literature reviews, key informant interviews, existing data on graduate career trajectories, and EWG deliberations informed the identification of career profiles for HSPR graduates and the competencies required to succeed in these roles. Stakeholder consultations were held to vet, refine, and validate the competencies.

**Principal Findings:**

The EWG reached consensus on six sectors and eight primary roles in which HSPR doctoral graduates can bring value to employers and the health system. Additionally, 10 core competencies were identified that should be included or further emphasized in the training of HSPR doctoral students to increase their preparedness and potential for impact in a variety of roles within and outside of traditional academic workplaces.

**Conclusion:**

The results offer an expanded view of potential career paths for HSPR doctoral graduates and provide recommendations for an expanded set of core competencies that will better equip graduates to maximize their impact on the health system.

Health services and policy research (HSPR) in Canada has grown significantly as a discipline over the past two decades, driven by an increasingly sophisticated demand for evidence‐informed insights to support changing delivery models and health system goals (Canadian Health Services and Policy Research Alliance 2015). Alongside this growth has come HSPR training programs that produce an increasing number of doctoral graduates equipped with sophisticated research and analytic skills to generate evidence in how to organize, fund, and deliver services (Grudniewicz et al. 2014; IHSPR [Institute of Health Services and Policy Research] 2016).

Like most doctoral programs, Canada's current HSPR programs have been designed to prepare graduates for traditional academic careers. The HSPR PhD curriculum concentrates largely on deepening knowledge and developing skills important in an academic workplace, focusing primarily on research methods. The career preferences of our doctoral students mirror those of Canadian doctoral students in general; the majority of students (65 percent overall in Ontario, 86 percent in the humanities) commence their PhD studies with a career in academia as their primary objective (Edge and Munro 2015).

Recently students and their supervisors have started to view nonacademic career paths as viable and valuable option. Students want to utilize their knowledge and skills to promote health system improvement, and increasingly view careers in health system settings as an effective way to make an impact. As health policy and delivery organizations in Canada work toward becoming learning organizations that harness data and evidence to inform policy, planning, and delivery, they are increasingly seeking embedded research talent. The delivery system scientist and learning health system researcher roles that are more common in the United States are beginning to pop up in Canadian hospitals. A number of federal and provincial governments now have embedded research, evaluation, data science, and behavioral insights units with managerial and leadership positions that prefer a PhD graduate. Health professional associations and provincial quality councils have departments devoted to research, data, and quality improvement. At least one HSPR doctoral program has taken strides to create partnered “hybrid” positions with health care organizations that are jointly funded by the university and the organization and include protected time for research and teaching as well as a focus on health system impact goals (Cockerill 2017).

However, many students are unaware of the full range of roles in which they can add value, and many supervisors continue to emphasize traditional academic careers as their gold standard. In addition, the work environments in health system and related organizations differ from the university setting in which HSPR PhD students are currently trained, and the skill set required to effect change and drive improvement within these organizations differs from those emphasized in PhD training (Reid 2016; Tamblyn et al. 2016). It is not surprising, then, to learn that in exploratory discussions leading up to the work described here, PhD graduates and employers spoke about their limited ability to promote change in health system organizations. Although employers recognized the potential value of adding PhD graduates to their teams, they also noted the need for a work‐in period as PhD graduates learned to adapt to the culture and demands of nonacademic environments.

Students’ changing career aspirations and their desire to contribute to health system improvement are starting to become better aligned with the current Canadian academic labor market in which only 18.6 percent of graduate trainees end up in tenure‐track academic positions (Edge and Munro 2015). This challenge is not unique to Canada, nor to HSPR: data from the Canadian Census show that between 1981 and 2007 the number of full‐time tenured or tenure‐track positions declined by 10 percent overall and by 23 percent for professors under the age of 35 (Desjardins 2012). In the United States, tenure‐track positions for biomedical PhDs fell from 34 to 26 percent of faculty complements between 1993 and 2012 (National Institutes of Health 2012). Despite this, data from Ontario indicate that enrolment in doctoral programs nearly doubled between 1999 and 2009 (Maldonado, Wiggers, and Arnold 2013). To align with these market trends and harness the full value that doctoral training can bring to health system improvement, our PhD programs need to modernize their approach and broaden their vision of existing and potential career options for their graduates.

In 2015, the Canadian Health Services and Policy Research Alliance (CHSPRA)—an alliance of 38 HSPR‐related organizations in Canada—identified the modernization of HSPR doctoral and postdoctoral training as a top priority. An Expert Working Group was established to develop a pan‐Canadian HSPR training modernization strategy. This manuscript describes the group's process and methods that resulted in the identification of an expanded range of academic and nonacademic roles suitable to HSPR PhD graduates, and a recommended expanded set of core competencies for further emphasis in HSPR doctoral training to support these roles. It builds on the work of Morgan, Orr, and Mah (2010) that produced a competency framework for HSPR master's students in Canada as well as on the framework of traditional HSR core competencies in the United Sates developed by Forrest et al. (2009) to introduce new concepts and recommendations that will help HSPR doctoral graduates add value and maximize their impact across a range of work environments and roles.

## Methods

Our work involved several stages described below, multiple sources of evidence, and significant stakeholder engagement throughout the process. A 16‐member Expert Working Group (EWG) led the work. This group was constructed to be broadly representative of the HSPR ecosystem in Canada and included leaders of doctoral training programs, health care decision makers, research funders, and graduate students. Members were drawn through a nominations process (including self‐nominations) at a national meeting that included all major HSPR funders, leaders of most of the country's doctoral training programs, and representatives of various health system organizations. Panel members appointed to ensure geographical and disciplinary balance and representation from the relevant stakeholder groups. The group was co‐chaired by two of the country's academic leaders (the director of a major HSPR training program and the director of a provincial health research funding organization), who had both previously held executive leadership roles in government.

### Phase 1: Identifying Career Profiles and Competencies Required to Succeed

The first phase of work involved the identification of career profiles for HSPR graduates and developing a draft set of competencies believed necessary to succeed and make an impact in these roles. It entailed two literature reviews; 19 key informant interviews; analysis of existing data on HSR funding levels and trends in Canada; where available, HSPR PhD production rates and career trajectories; and deliberations by the EWG.

Two initial searches of peer‐reviewed and gray literature were conducted using the updated scoping review methodology described by Levac, Colquhoun, and O'Brien (2010). The first examined developments in doctoral‐level training in general and in HSR PhD training in Canada and other countries. The second review examined the range of potential academic and nonacademic roles HSR doctoral graduates may occupy when they join the workforce, as well as the competencies required to excel in these roles (CHSPRA TMWG 2015a). Peer‐reviewed literature relating to these topics was sparse, as were data on employment opportunities and career trajectories for Canadian HSPR PhDs graduates, as 80 percent of universities do not track their graduates (Grudniewicz et al. 2014). Available data came primarily from Ontario and were later supplemented with data from Manitoba, but consultations with the EWG confirmed that the patterns observed in those provinces were broadly representative of the broader Canadian employment landscape.

Searches were conducted in MEDLINE and Google Scholar, were limited to the English language, and did not impose country or date restrictions. One member of the project team examined the full text of each article and the reference lists of those deemed relevant for additional studies, and extracted information from relevant articles and reports (*n* = 44) using a standardized form. The full EWG reviewed the extracted data and recommended additional reports and websites to assess for relevance.

Findings from the literature reviews and input from the EWG informed the development of a semi‐structured interview guide to elicit perspectives of key stakeholders regarding existing and emerging sectors and roles in which HSPR PhD graduates can add value, the key competencies required for success, and opportunities and challenges for the future of HSPR training. Interviews were conducted by one member of the project team with a purposive sample of 19 key informants from relevant stakeholders groups, including researchers, HSPR PhD training programs, public and private sector health system employer organizations, and current trainees.

### Phase 2: Drafting the Initial Strategy, Career Profiles and Core Competencies

A subgroup of the EWG (*n* = 4, including the co‐chairs) reviewed all available information. By triangulating the data and drawing on their own expert opinions, they drafted a summary document outlining potential options for a pan‐Canadian training modernization strategy, including a preliminary list of sectors and primary roles suitable for HSPR PhD graduates, and a draft set of eight core competencies required for success in these roles. They assessed the similarities and differences between their recommended set of core competencies and other HSR core competency frameworks (Forrest et al. 2009; Morgan, Orr, and Mah 2010) and reviewed each primary role against the set of core competencies to determine whether any essential competencies had been overlooked. Underlying the group's deliberations and assessment was an explicit commitment to the importance of rigorous scholarly training. There was unanimous agreement that the competencies should enhance, not detract, from academic excellence and that they should prepare PhD graduates to contribute their skills and talents in a range of sectors and roles including, but not limited to, the traditional and embedded researcher roles targeted by other HSR competency frameworks (see Forrest et al. 2009, 2018).

The subgroup developed five criteria to use for assessing the potential options for a pan‐Canadian strategy: (1) potential impact on HSR training capacity and outcomes, (2) pan‐Canadian scalability, (3) flexibility to accommodate different student phenotypes (e.g., students with different education and training backgrounds and career objectives), (4) economic feasibility, and (5) conduciveness to a collective impact approach (in which value‐add and economies of scale could be achieved through pan‐Canadian collaboration).

The full EWG met to consider the options using the assessment criteria and to review the career profiles and draft competencies. The group reached a consensus to recommend six strategic directions, as well as six sectors, eight primary roles, and eight core competencies believed essential for success.

The co‐chairs presented the EWG's recommendations at the May 2015 CHSPRA Annual General Meeting and, receiving endorsement from all member organizations, produced an initial pan‐Canadian strategy document (CHSPRA TMWG 2015b).

### Phase 3: Vetting and Validating the Strategy

An invitational HSPR Training Modernization Symposium was hosted in March 2016 to vet and validate the strategy. One hundred leaders of HSPR training programs, executives and clinicians from health system organizations, research funders, trainees, and international experts were convened to review, discuss and, if needed, refine the strategy and the proposed core competencies, and to prioritize the strategic directions for initial action. The strategy was endorsed and two strategic directions were prioritized: (1) creating experiential learning opportunities within health system organizations, and (2) enriching traditional academic core competencies with professional competencies. The eight core competencies were also endorsed and expanded to include two others, bringing the final set to 10.

This manuscript and the findings detailed below focus on two central components of the training modernization strategy: the career profiles and the enriched core competencies (see McMahon et al., forthcoming, for the results of the full strategy and implementation plan).

## Principal Findings

The EWG identified diverse sectors and roles in which HSPR PhD graduates can add value and contribute to improved health system performance (Table 1). It is important to note that the six sectors and eight roles are not mutually exclusive: an individual's career profile could involve multiple transitions among sectors and roles over time, or simultaneous employment in different sectors and roles.

**Table 1:** Sectors and Primary Roles for HSPR Doctoral Graduates Entering the Workforce

Sectors	1. Academic	2. Government	3. Not‐for‐Profit
	Includes universities and hospital research institutes	Includes ministries, agencies, boards, and commissions	Includes NGOs, health charities and providers/hospitals	
	4. Independent Research Organization	5. For Profit	6. International Agency/Organization	
	Includes organizations like The Conference Board of Canada	Includes consulting, biotech, pharmaceuticals, and providers	Includes international agencies and organizations based inside and outside Canada	
Roles	1. Research	Conducting applied research in various environments (academic and nonacademic).	Traditional HSPR Competencies + Leadership, Mentorship & Collaboration; Project Management; Interdisciplinary Work; Dialog & Negotiation	Required Competencies
	2. Clinician Researcher	Conducting research while working as a practicing health care provider		
	3. Management of Research Activities	Planning, organizing, or managing research activities.		
	4. Teaching and Mentorship	Mentorship and teaching activities in a formal and informal way. These activities are focused on trainees, researchers, colleagues, patients, policy makers, and others.		
	5. Management of Research Funding Programs	Management or leadership of research funding programs.	Professional HSPR Competencies + Understanding Health Systems & Policy‐Making Processes; Analysis & Evaluation of Health‐related Programs and Policies; Analysis of Data, Evidence & Critical Thinking; KT, Communication and Brokerage	
	6. Development and Evaluation of Policies and Programs	Working inside an organization in a primary research, evaluation, quality improvement or policy role focused on policies and program development and evaluation.		
	7. Management of Knowledge Translation and Brokering	Working in a direct knowledge translation role translating research into policy, programs and services, includes capacity development around research, evidence use, and quality improvement within organizations.		
	8. Management/Administration of Health or Health‐Related Organization	Working as a manager or leader in an organization without a primary research role, including in the for‐profit, not‐for‐profit, and government sectors.		

**Figure 1:**
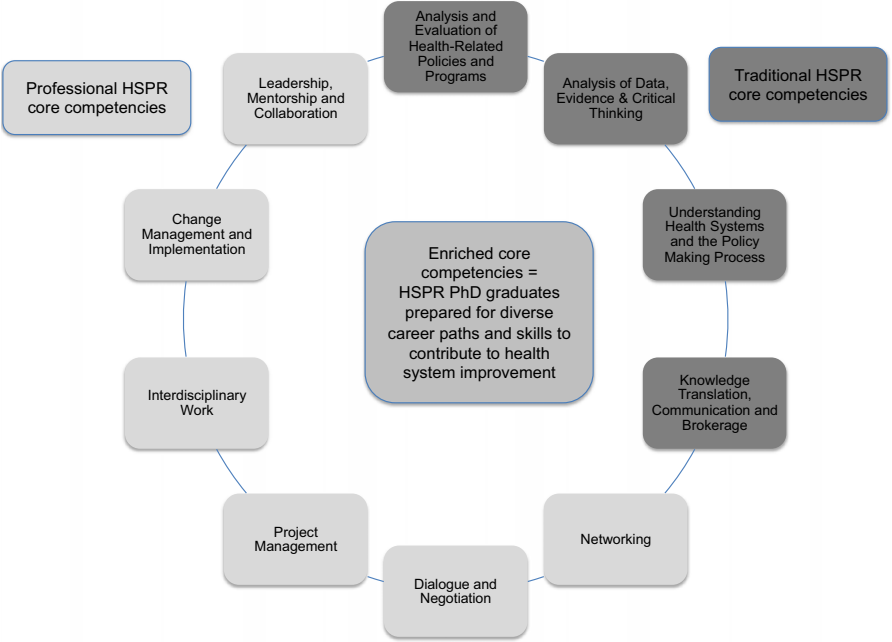
Enriched Core Competencies [Color figure can be viewed at http://wileyonlinelibrary.com]

The EWG's proposed suite of 10 enriched core competencies is illustrated in Figure 1. As shown in Table 1, the traditional and professional competencies are relevant and transferrable across the majority of sectors and roles. The traditional academic core competencies (e.g., analysis of data, evidence, and critical thinking; understanding and comparing health systems and policy‐making processes) are relevant beyond the academic sector and the researcher role. Similarly, the new professional competencies, such as leadership and project management, will add value in both academic and nonacademic sectors. This is not surprising given that the EWG set out with the deliberate intention of identifying competencies that would prepare students to succeed in a wide variety of sectors and roles, including both traditional researcher roles and manager or executive leader roles in a health care delivery organization, and in aspirational roles that may emerge as Canada's labor market evolves to better harness PhD talent for improved health system performance.

The traditional HSPR core competencies are not new to Canada and are already largely addressed within contemporary HSPR curricula. They are similar to HSR core competencies in the United States, which tend to emphasize the skills required to succeed as a *researcher* in an academic or embedded setting (Forrest et al. 2009, 2018). An examination of existing competency frameworks indicates that professional competencies tend to receive low attention and, when they are emphasized, it is with reference to a researcher role.

Figure 1 also shows the professional competencies that the EWG recommended for inclusion or increased emphasis in the training of HSPR doctoral students. A definition and rationale for each competency is provided below.

### Competency 1: Analysis and Evaluation of Health‐Related Policies and Programs

#### Description

The ability to ask relevant research questions and effectively carry out formative and summative evaluation with meaningful outcome measures and strong links to organizational improvement and planning, includes theoretical knowledge, technical skills, contextual awareness, and collaboration skills.

#### Rationale

Governments and health care organizations must increasingly make difficult decisions about the financing, organization, and delivery of health care. The analysis and evaluation of health‐related policies and programs calls for a systematic approach that is rooted in an ability to effectively carry out formative and summative evaluation, increasingly using a participatory approach involving patients, clinicians, and/or policy makers to ensure measurement of relevant and meaningful outcomes, and with strong links to organizational improvement and planning. This requires technical competency, contextual awareness, and skills in interdisciplinary collaboration.

### Competency 2: Critical Analysis of Data and Evidence

#### Description

The ability to collect, analyze, interpret, and use a wide range of data and to reflect critically on and iteratively incorporate theory and research evidence in order to clarify issues, ask relevant questions, frame options, identify implementation considerations, and communicate findings in both academic and nonacademic settings, includes qualitative and quantitative data, including big data, electronic health record data, administrative data, economic data, and patient‐reported data.

#### Rationale

Our increasingly complex health systems require sophisticated skills in data analytics and mixed‐methods approaches to problem solving. The ability to collect, analyze, and interpret a wide range of data to inform improvements in the way care is financed, organized, and delivered is essential. Graduates must be able to interpret different kinds of data, reflect critically on theory and on research evidence, and apply their knowledge iteratively to the workplace in order to probe complex problems from multiple directions, frame options, and identify evidence‐informed considerations for implementation.

### Competency 3: Understanding Health Systems and the Policy Making Process

#### Description

Excellent knowledge of the Canadian and international health care systems from both academic and real‐world perspectives, including how systems are organized, financed, and managed, and how care is funded and delivered, includes knowledge of the health policy‐ and decision‐making process and the external factors that influence decisions.

#### Rationale

An excellent understanding of the health care system—including how it is financed, organized and delivered and the major stakeholders—is essential foundational knowledge for any HSPR graduate, both in terms of conducting research in complex systems and ensuring that evidence gets translated into improved policies, services, and products.

### Competency 4: Knowledge Translation, Communication, and Brokerage

#### Description

The ability to integrate evidence into real health and health system improvements, to use multiple methods of communication, and to communicate appropriately and effectively with different audiences.

#### Rationale

The evaluation of effective research capacity extends beyond traditional measures, such as numbers of publications and successful grants, to the impact of research (Cooke 2005). Ensuring that evidence has impact requires the ability to translate knowledge into better decisions. It also requires the ability to use multiple methods to communicate appropriately with different audiences and stakeholder groups.

### Competency 5: Interdisciplinary Work

#### Description

The ability to effectively use and combine methods and insights from multiple disciplines (e.g., humanities, social sciences, management, epidemiology, medicine, etc.), as well as the ability to engage and collaborate with partners and stakeholders from a wide range academic disciplines, professional backgrounds, and institutional contexts.

#### Rationale

The ability to effectively draw on methods and insights from multiple academic disciplines is key to tackling some of our health systems’ most complex issues. For example, a robust approach to patient safety can require expertise in health services research as well as understanding of concepts and approaches from other fields such as epidemiology, improvement sciences, organizational behavior, psychology, political science and engineering, without necessarily mastering the canon of each of these fields.

### Competency 6: Leadership, Mentorship, and Collaboration

#### Description

The ability to organize, lead, and support diverse teams to achieve a specific outcome, includes skills in innovative and adaptive thinking, building and motivating teams, setting direction, fostering self development and the development of others, and collaboration.

#### Rationale

As workplaces recognize the importance of research skills, PhD graduates are increasingly finding themselves in new roles requiring leadership, mentorship, and collaboration. While PhDs are often well versed in collaborative skills, other traditional PhD characteristics—such as thoroughness and exhaustive analysis—can prove a hindrance to collaboration, rapid decision‐making, and the efficient delivery of work. Leadership and mentorship skills that involve organizing and supporting groups of diverse professionals to work together and achieve a specific outcome in a defined time period are imperative to solving complex health system challenges.

### Competency 7: Networking

#### Description

The ability to develop and maintain productive relationships within and outside of academia across the health system.

#### Rationale

The ability to develop and maintain productive relationships within and outside of academia is a critical skill required for effecting health system change. Networking supports the identification of individuals and organizations able to support evidence‐informed action, facilitates the creation of coalitions to support change, and provides support for knowledge transfer. More generally, it supports collaboration and knowledge sharing across sectors to improve the health system.

### Competency 8: Project Management

#### Description

The ability to plan, coordinate, and organize all stages of a project in order to optimize resource use and achieve key objectives in both academic and nonacademic environments.

#### Rationale

Project management is an essential competency for getting things done. It is the ability to plan and execute all stages of a project, from initiation through to implementation and knowledge translation. As we strive to ensure our policies, programs and services advance the Triple Aim objectives of improved patient experience, improved population health, and reduced health care cost (Berwick, Nolan, and Whittington 2008), and the need for sophisticated project management approaches is critical. Understanding and applying the principles of project management to research and nonresearch activities can help to ensure projects are completed to achieve their desired impact and add value to our health system.

### Competency 9: Dialog and Negotiation

#### Description

The ability to work toward win‐win outcomes, including understanding others’ perspectives and how to respond, includes skills in impact and influence, empathy, negotiation, and effective communication.

#### Rationale

Effective dialog and principled negotiation are essential for promoting change in complex systems that typically involve multiple and diverse sectors, stakeholders and priorities. Fostering dialog and the open exchange of ideas, empathizing with the perspectives of others and understanding how to respond, and managing conflict and achieving resolution are essential skills for building trust, fostering collaboration, and achieving results.

### Competency 10: Change Management and Implementation

#### Description

The ability to plan, manage, and implement change, including to communicate a clear vision and rationale for change; to mobilize people and lead organizations through change; to manage and implement successful transitions; and to evaluate and report on change.

#### Rationale

Effective workplaces in challenging environments must experiment, evolve, and innovate continuously. Policy makers often express frustration at the dearth of evidence regarding relevant strategies for implementing change. Skills in communicating a clear vision and rationale for change, leading people and organizations through change, managing and implementing successful transitions, and evaluating and reporting on change will be immensely valuable in any learning organization.

## Discussion

The challenges facing health systems worldwide are extensive, multifaceted, and complex. The convergence of demographic shifts, accelerating technological innovation, and growing pressures to simultaneously improve quality and efficiency and control costs have created a pull for sophisticated research expertise and innovative thinking to help design, implement, and evaluate evidence‐informed solutions. In addition, the concept of the learning health system is spreading, and health care organizations are working to harness the power of data, decision sciences, and patient engagement to drive continuous improvement. This creates a growing need for researchers with a wider array of skills like leadership, communication, and management (Tamblyn 2014; Reid 2016; Forrest et al. 2018) as well as for health care leaders with a commitment to evidence‐informed continuous improvement (Morain, Kass, and Grossmann 2017). Well‐prepared PhD graduates can make important contributions to health policy and system transformation, whether as embedded researchers or as health system leaders.

Current HSPR doctoral training programs in Canada equip students with a deep understanding of the health care system, skills in developing and applying research theories and methods, and a range of technical and analytic skills appropriate for academic careers. These skills are also valuable in other sectors and roles, but they are insufficient on their own for jobs in nonacademic markets, learning health systems, or hybrid jobs that span both the academic and the nonacademic worlds. The broader skills required for success in these other settings and roles are not yet emphasized in HSPR PhD curriculum. However, pockets of innovation exist upon which to learn and build. In Canada, Quebec's Transdisciplinary Research Training Program in Public Health Interventions was introduced in 2003 to link science and practice by embedding doctoral and postdoctoral trainees in public health organizations (Paradis et al. 2017), and the Capacity for Applied and Developmental Research and Evaluation (CADRE) Regional Training Centres (RTCs) that were funded from 2000 to 2010 made significant contributions to building capacity for applied HSPR (Conrad 2008)—but these were both grant funded programs with fixed funding terms. In the United States, the DrPH, an advanced professional degree common in the United States but not yet in Canada, emphasizes leadership and management but does not provide the depth of training in research theory and methods that the PhD degree offers. The biomedical and applied sciences disciplines have already realized the value that comes from blending strong research training with enhanced professional skills and routinely offer opportunities for experiential learning and professional skills development so that PhD graduates can successfully pursue multiple career options.^1^

It is now time to build on the ideas inspired by innovative programs like these and make enhanced competency development mainstream elements of HSPR doctoral training in Canada. These ideas are not currently mainstream, although a recent survey of the deans of 20 of Canada's HSPR PhD training programs revealed that the six professional competencies recommended through this work are rarely included in HSPR PhD curricula and, when they are, it is typically only implicitly and indirectly in course materials or on an elective basis. For example, when asked on a scale of 0 to 7 the extent to which their PhD program provides training in change management, the average self‐assessed rating was 2.9 (IHSPR 2017). Although not yet mainstream, there appears to be enthusiasm to move down this path: 100 percent of the survey respondents indicated that they felt their HSPR PhD program would benefit from a pan‐Canadian curriculum with supporting course materials to help ensure their doctoral students have access to training in the enriched competencies.

Given the current trend toward producing more PhDs in Canada and the evidence that PhDs increase a country's commercial and innovative power (Conference Board of Canada 2015), Canadian educators should work to modernize and enhance our PhD programs. Improved training can yield more marketable graduates who can combine research, insight, and leadership in a variety of roles and workplaces. The competencies outlined here reflect many of the skills employers are looking for today. It is increasingly being recognized that the enhancement of traditionally strong PhD skills with leadership development and other transferrable skills brings with it new opportunities and careers outside academia, and that these enhancements do not invalidate or interfere with rigorous grounding in more scholarly competencies (Forster 2015).

The enriched core competency framework outlined in this manuscript will add value to HSPR PhD graduates, to the organizations employing them, and to the health system more broadly by blending rigorous academic training with professional skills development. Although there is no empirical work to support the generalizability of our enriched core competencies, similarities between Canadian and U.S. traditional core HSPR competencies (Forrest et al. 2009) and the recognition of leadership and management competencies in the learning health system researcher competencies (Forrest et al. 2018) is promising and suggestive of the potential for utility beyond Canada's HSPR programs.

As the enriched core competencies are implemented across training programs in Canada, it will be important for students, early in their training programs, to understand the full range of options for future employment available to them as well as the key attributes that nonacademic employers are seeking (Lee et al. 2015). Although the hiring criteria for faculty positions may focus primarily on a candidate's academic and scientific record, hiring criteria for nonacademic careers tend to emphasize transferable skills and experience. Beyond an expanded set of competencies, students who are interested in nonacademic careers should be given opportunities to gain practical experience, to develop relationships and collaborate with leaders of health system organizations, and to witness how research skills can contribute to an organization's performance. This may also help address the negative perception that PhDs are simultaneously over‐ and under‐qualified (Callier and Vanderford 2015).

Moving forward, improvements in tracking our graduates could also help to better delineate the true job market for HSPR PhDs, to more accurately inform students of their career options early in their training and to further refine our understanding of the competencies required of graduates in the workplace. In addition, the expansion of experiential learning opportunities, field immersion, and embedded fellowships may help students to transition more easily to the nonacademic job market after they graduate (Lee et al. 2015). A new fellowship offered by Canada's Institute of Health Services and Policy Research—the Health System Impact Fellowship—is providing dedicated funding and national recognition for highly qualified postdoctoral fellows to pursue experiential learning opportunities and enriched competency development within health system organizations (see McMahon et al., forthcoming, for details).

The health care sector plays a prominent role in the economy. It is an economic driver, a source of jobs and income, and a producer of health and well‐being. It is an incredibly complex sector with high stakes—people's health. PhD graduates in HSPR have acquired the in‐depth skills and knowledge about how to analyze what works, for whom, in what settings and why to improve the health sector's performance. They do not yet, however, received training in competencies that will enable them to harness these skills at the coalface of health policy and delivery to effect change and make an impact. HSPR doctoral programs require modernization to meet the needs of evolving healthcare systems. This work builds on existing competency frameworks to propose an expanded set of HSPR competencies for incorporation into doctoral training. These expanded competencies are intended to allow graduates to effectively contribute to the improvement of HSPR across a variety of academic and nonacademic settings and roles, and maximize their impact across the health system. Close assessment of how the competencies are operationalized by Canada's training programs, how students’ career preparedness and career trajectories are affected, and whether there are additional sectors and roles that should be incorporated within our career map will inform future iterations of Canada's training modernization efforts.

## Supporting information

Appendix SA1: Author Matrix.

## Note

1 See Canada's Mitacs Elevate and Accelerate programs, the European Commission's Marie Skłodowska‐Curie actions program, and the University of Toronto's biochemistry graduate professional development course for examples of programs that aim to prepare PhDs for careers in industrial innovation.
